# Long-term effect of the perindopril/indapamide/amlodipine single-pill combination on left ventricular hypertrophy in outpatient hypertensive subjects

**DOI:** 10.1016/j.biopha.2019.109539

**Published:** 2019-10-15

**Authors:** Alberto Mazza, Danyelle M. Townsend, Laura Schiavon, Gioia Torin, Salvatore Lenti, Ciro Rossetti, Gianluca Rigatelli, Domenico Rubello

**Affiliations:** a ESH Excellence Hypertension Centre, Internal Medicine Unit, S. Maria della Misericordia General Hospital, AULSS 5 Polesana, Rovigo, Italy; b Department of Drug Discovery and Biomedical Sciences, Medical University of South Carolina, USA; c Unit of Internal Medicine, S. Maria della Misericordia Hospital, AULSS 5 Polesana, Rovigo, Italy; d Unit of Internal Medicine C, Department of Medicine, University of Verona, Verona, Italy; e Internal Medicine Unit, S. Donato General Hospital, Arezzo, Italy; f Interventional Cardiology Unit, Division of Cardiology, S. Maria della Misericordia Hospital, AULSS 5 Polesana, Rovigo, Italy; g Department of Nuclear Medicine, Radiology, Neuroradiology, Medical Physics, Clinical Laboratory, Microbiology, Pathology, Trasfusional Medicine, Santa Maria della Misericordia Hospital, Rovigo, Italy

**Keywords:** Single-pill combination, Hypertension, Left ventricular hypertrophy, Regression

## Abstract

**Background:**

Most antihypertensive drugs used in monotherapy or in combination therapy reduce the left ventricular mass index (LVMI). However, little is known about the effects on LVMI of a triple fixed-dose combination (TFC) therapy, containing in a single pill an angiotensin-converting enzyme inhibitor (ACEI), a diuretic and a calcium channel blocker (CCB).

**Methods:**

In this prospective open-label study, 92 patients with essential hypertension were randomized to treatment with a TFC of perindopril/indapamide/amlodipine at different doses or a triple free combination therapy (FCT) including ACEI/diuretic/CCB. Office blood pressure (BP) measurement, 24 h-ambulatory BP monitoring and echocardiography were performed at baseline and during a 14-month follow-up. The BP variability (BPV) over 24 h was calculated as ± standard deviation of the daytime systolic BP. Differences between office and monitored BP and LVMI were evaluated by ANOVA for repeated measures.

**Results:**

A significant BP-lowering effect was observed for both treatments. At follow-up, BPV was reduced in both the treatment groups *vs*. the baseline (14.0 ± 1.5 *vs.* 17.0 ±1.8 and 16.2 ± 2.1 *vs.* 17.6 ± 2.3, respectively), but it was lower in the TFC *vs*. the FCT group (14.0 ± 1.5 *vs.* 16.1 ± 2.2, P < 0.05). LVMI was lower in both the treatment groups, but the change was greater for TFC *vs.* FCT (−8.3 ± 4.9% *vs.* −2.0 ± 2.1%, P < 0.0001). Left ventricular hypertrophy (LVH) regression was greater in the TFC *vs*. the FCT group (43.5% *vs*. 30.4%, P < 0.05).

**Conclusions:**

Independently of BP values achieved, the antihypertensive TFC therapy was more effective than FCT in LVMI reduction and LVH regression, possibly related to drugs’ intrinsic properties and to BPV modulation.

## 1. Introduction

In arterial hypertension, left ventricular hypertrophy (LVH) is a compensatory process representing an initial adaptation to increased ventricular wall stress [1]. However, LVH is the first step toward the development of overt clinical disease, as observed both in clinical and epidemiological studies, including also elderly patients. A strong relationship between LVH at baseline examination and the risk of morbid or mortal cardiovascular events has been reported [2].

According to electrocardiographic criteria, the prevalence of LVH is quite low in the general population increasing to 7–40% in hypertensive population [2].

In addition, an echocardiographic screening revealed that the prevalence of LVH could reach over 50% of subjects older than 65 years [3]. The relationship between echocardiographic left ventricular mass index (LVMI) and clinic blood pressure (BP) is usually weak, while the 24 h ambulatory blood pressure monitoring (ABPM) has shown a much closer correlation between LVMI and average daytime BP values [4].

Observational and prospective studies examined the potential clinical benefits of LVMI reduction and demonstrated that changes in LVMI during treatment may have an important prognostic role in hypertensive subjects [5].

Indeed, subjects who failed to achieve LVH regression or in whom LVH developed during the follow-up were much more likely to suffer morbid events than those in whom LVH regressed or never developed [6,7].

This confirms that LVMI changes during antihypertensive treatment were the most important factor related to the occurrence of cardiovascular fatal and non-fatal events [8].

Many studies have suggested that LVH regression may be more rapidly or more completely obtained through specific classes of antihypertensive drugs, such as ACE-inhibitors (ACEIs), angiotensin receptor blockers (ARBs), and calcium channel blockers (CCBs), as monotherapy [9–11] or single-pill therapy [12–14].

However, little is known about the effects on LVMI changes and LVH regression of a triple fixed-dose combination (TFC) therapy containing in a single pill an ACEI, a diuretic and a CCB.

The aim of this study was to examine the long-term effect of a TFC of the antihypertensive drugs perindopril/indapamide/amlodipine on LVMI lowering and LVH regression in outpatient hypertensive subjects, previously uncontrolled with a dual fixed combination of a renin-angiotensin-aldosterone system (RAAS) inhibitor and a diuretic.

## 2. Materials and methods

This interventional study enrolled patients without history of cardiovascular events and with grade-2 essential hypertension (*i.e.* office systolic BP (SBP) < 140 mmHg and diastolic BP (DBP) < 90 mmHg), previously uncontrolled with a dual fixed-combination therapy including a RAAS inhibitor plus a diuretic. Exclusion criteria were: severe hypertension (office SBP ≥ 180 mmHg or DBP ≥ 110 mmHg), secondary hypertension, neoplastic or hepatic diseases, chronic heart or renal failure, positive history or clinical signs of ischemic heart disease, severe obesity (body weight > 150% of the ideal), disabling diseases (*e.g.* dementia or inability to cooperate), pregnancy or breast-feeding. Subjects were enrolled between October 2015 and June 2016 in our Hypertension Centre, and gave informed consent for participation to the study. Local Ethics Committee and institutional review boards approved the protocol. The study was conducted in accordance with ICH Harmonized Tripartite Guidelines for Good Clinical Practice and the Declaration of Helsinki Principles. Subjects were free to discontinue the study at any time and for any reason, and the treatment was stopped if a serious adverse event occurred.

### 2.1. Study design

Study recruiting methods and the procedures for the baseline examinations and the follow-up have been described in detail elsewhere [15]. The patients were switched to treatment with a TFC of perindopril/indapamide/amlodipine given once daily and compared to a control group of hypertensive patients taking a free combination therapy (FCT) of three antihypertensive drugs, including a RAAS blocker, a diuretic and a CCB. TFC patients were age- and gender-matched by the case-to-case method with the FCT control group. After the initial screening and a run-in period of 4 weeks, patients treated with FCT were assigned to receive in the morning one tablet once daily of perindopril/indapamide/amlodipine at different doses (5/1.2/5, 10/2.5/5, 10/2.5/10 mg daily) for 4 months. The different doses used in the fixed-combination arm depended on the degree of baseline BP values, however in the course of the study all the subjects were treated with the highest tolerated doses to reach BP control. In this respect, in clinical trials a careful choice of the appropriate dose medication is fundamental to avoid dose-related side effectscausing the dropoutfrom treatment and leading to failure of BP control. The subjects in the FCT groups were only in part randomized to standardized classes of antihypertensive agents, as 35% of them were intolerant to ACE-inhibitors and 30% to amlodipine treatment. Equivalent doses of the antihypertensive agents were used within the FCT groups and compared to the TFC arm. Office and 24 h BP values were evaluated at baseline and at the 1st, 4th and 14th month of follow-up. The aim of this study was to evaluate the efficacy of the TFC therapy *vs*. FCT in LVMI reduction and in LVH regression, with a target BP defined as SBP < 140 mmHg plus DBP < 90 mmHg in office, and as SBP < 130 mmHg plus DBP < 80 mmHg at 24 h-ABPM.

### 2.2. Data collection

At baseline, office BP (diastolic Korotkoff phase 5) was measured in triplicate in the lying position using a mercury sphygmomanometer at 10-minute intervals, taking special care to avoid any terminal digit preference [16]. To minimize potential white-coat effects, the average of the last two clinostatic measurements was taken as BP. Heart rate was also measured at the same time. Pulse pressure (PP) was defined as the difference between SBP and DBP. Hypertension was defined as SBP ≥140 mmHg or DBP ≥90 mmHg. Clinic hypertension was confirmed in all subjects by 24 h ABPM, detected through an oscillometric device (TM-2430, Takeda, Japan) applied to the non-dominant arm [17]. BP variability (BPV) was calculated as the standard deviation (SD) of average SBP and DBP day-time values over the 24 h. Body mass index (BMI) was calculated as the ratio of weight (in kilograms) to squared height (in meters). Subjects were classified into never and current (≥1 cigarette daily) smokers. Safety parameters monitoring included serum potassium and creatinine. Fasting serum creatinine (in mg/dl) was measured using the alkaline picrate-kinetic method of Jaffè by means of an auto-analyzer (Hitachi Modular P, Roche diagnostic, USA). Estimated glomerular filtration rate (ml/min/1.73 m^2^) was calculated from serum creatinine (mg/dl) using the formula of Cockroft and Gault [18]. Adherence to medication was measured through indirect methods, such as patient self-reports and pill counts.

### 2.3. Echocardiography

Hypertensive patients were imaged, and two different observers, blinded to treatment taken by patients, analyzed the data. M-mode and 2-dimensional echocardiography was performed with an IE33 system (Philips Medical System, Bothell, WA, United States), consistently with the recommendation of the American Society of Echocardiography [19]. Septal and posterior wall thicknesses and left ventricular (LV) end-diastolic dimensions were measured, and LV ejection fraction (in %) and LV mass were determined. LV mass was estimated by the Devereux formula [20], and then normalized for body surface area (LVMI, in g/m^2^). LVH was defined as a LVMI value ≥115 g/m^2^ for men and ≥95 g/m^2^ for women. Delta LVMI (in %) corresponds to LVMI change at follow-up *vs*. baseline. LVH regression at the follow-up was defined as LVMI values < 115 g/m^2^ for men and < 95 g/m^2^ for women.

### 2.4. Statistical analysis

Continuous variables were averaged, expressed as mean ± SD, and compared using the analysis of variance (ANOVA) and the Bonferroni’s post-hoc test. Comparison between categorical variables was done using the χ^2^ test. ANOVA for repeated measures compared the changes of different BP-related parameters and of serum lipids at baseline and at the follow-up visits. Differences between continuous variables were evaluated in the two study groups with the Tukey’s post-hoc test. All statistical analyses were performed using the SPSS package version 18.0 for Windows (SPSS, Chicago, IL, USA). The null hypothesis was always rejected for *P* < 0.05.

## 3. Results

The baseline characteristics of the TFC and FCT treatment groups are summarized in Table 1. Mean age of all patients was 60.8 ± 12.1 years (59.4 ± 11.5 years for men and 63.8 ± 13.9 years for women, P < 0.001). LVH prevalence did not significantly differ between the two study groups (71.7% *vs.* 68.5%, respectively, P = 0.63). In the FCT group, the proportion of patients taking ACEIs or Angiotensin-II receptor blockers (ARB) was 65% and 35%, respectively. In detail, among patients on ACEIs, 40% took ramipril, perindopril, 25% enalapril and 5% lisinopril. Among patients on ARBs, 35% took valsartan, 35%, olmesartan, 20% telmisartan, 15% losartan and 5% other ARBs. For diuretics, the proportion was 75% hydrochlorothiazide and 25% indapamide, while for CCBs, the proportion was 70% amlodipine, 15% barnidipine, 10% lacidipine and 5% other CCBs.

**Fig. 1.**
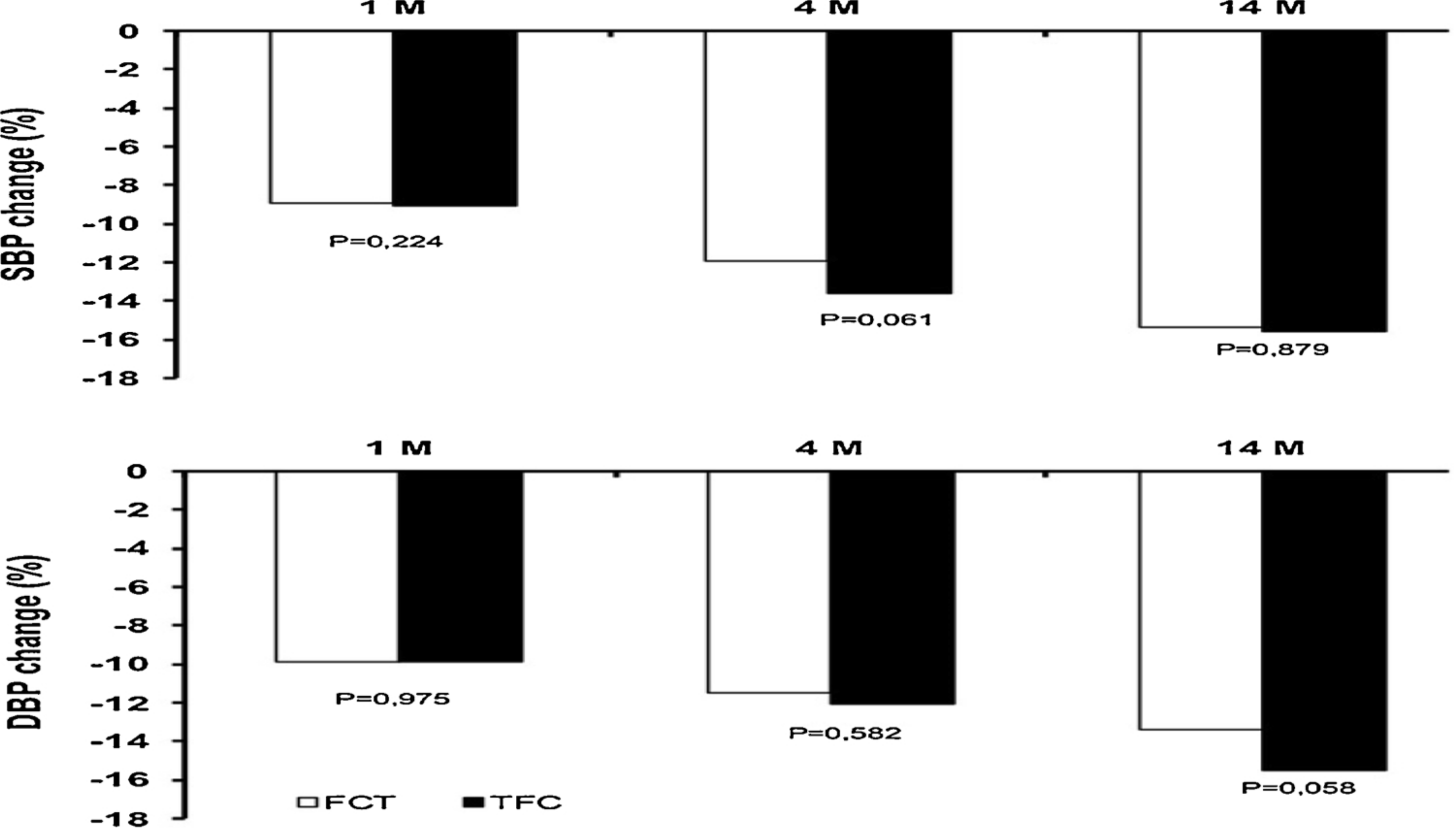
Office systolic blood pressure (SBP) and diastolic blood pressure (DBP) changes from baseline at the 1st, 4th and 14th month (M) of follow-up in the triple free combination therapy (FCT) and in the triple fixed-dose combination (TFC) groups. P value: follow-up *vs.* baseline.

During the follow-up, a significant office BP lowering effect was observed in both the TFC and FCT groups, with a greater effect for the TFC therapy (not significant *vs.* FCT, Fig. 1). A significant reduction in 24 h-ABPM values was also observed at the end of follow-up in both the treatment groups (Tables 2 and 3). In more detail, at the 1^st^ month of follow-up a significant reduction of 24 -h, daytime and night-time SBP and PP was found in the TFC but not in the FCT group, while at the 4^th^ month and at the end of follow-up the BP values were not different between the two study groups. 24 h-ABPM DBP and heart rate were unchanged during the entire follow-up. At the end of the 1^st^ month of follow-up, target office BP was reached by 70.5% of patients on TFC therapy and by 66.4% of patients on FCT (P = 0.21), at the 4^th^ month by 75.6% and 71.2% of patients, (P = 0.34) and at the 14^th^ month 77.1% and 72.4% of patients (P = 0.16), respectively. When target 24 h-ABPM was evaluated, the response rate to treatment was significantly higher in the TFC than in the FCT group at the 1^st^ month of follow-up (45.3 *vs*. 32.8%, P < 0.05), at the 4^th^ month (64.8% *vs.* 46.9, P < 0.05), and at the end of follow-up (69.7 *vs.* 53.2, P < 0.05).

**Fig. 2.**
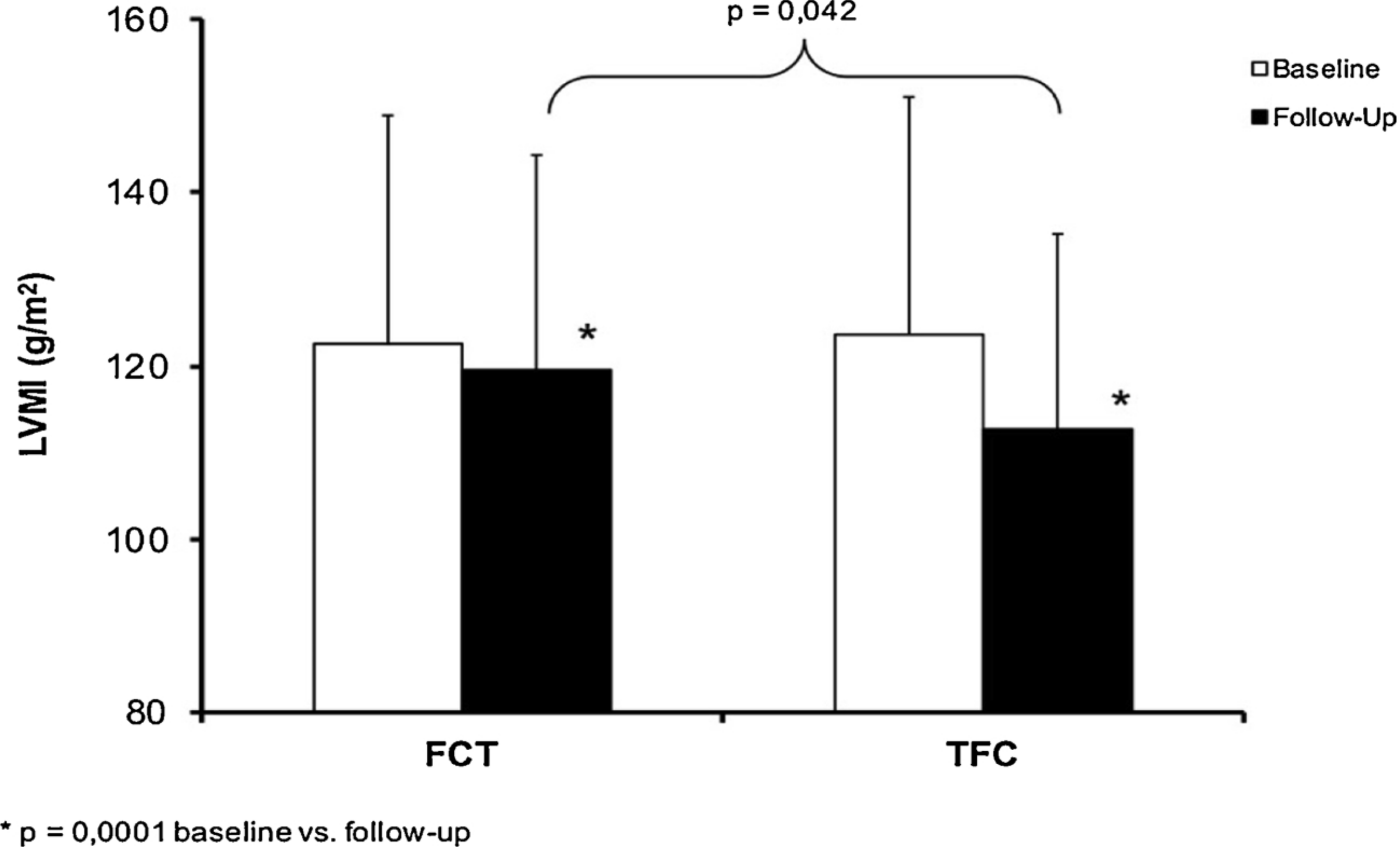
Left ventricular mass index (LVMI) changes from baseline to the end of follow-up in the triple free-combination therapy (FCT) and in the triple fixed-dose combination (TFC) groups. basend of follow-up *vs.* baseline.

At the end of follow-up, the BPV was reduced in both groups compared with baseline (14.0 ± 1.5 *vs.* 17.0 ± 1.8 in the TFC group and 16.2 ± 2.1 *vs.* 17.6 ± 2.3 in the FCT group), but it was significantly lower in the TFC than in the FCT group (14.0 ± 1.5 *vs.* 16.1 ± 2.2, P < 0.05). A similar result was observed for LVMI, that was lower in the TFC than in FCT group (Fig. 2), with a significant delta change difference of −8.3 ± 4.9 *vs.* −2.0 ± 2.1% (P < 0.0001) in the former compared with the latter. LVH regression was greater in the TFC than in FCT treatment group (43.5% *vs*. 30.4%, P < 0.05), without any gender difference. Mean change in serum potassium values from baseline to end of the follow-up significantly decreased in the TFC group (4.36 ± 0.22 *vs.* 3.89 ± 0.21, P < 0.001) but not in the FCT group (4.17 ± 0.31 *vs.* 4.10 ± 0.19, P > 0.05). Three patients withdrew from the research in the TFC group (2 for ankle oedema and 1 for cough) and 2 in the FCT group (1 for hypotension and 1 for dizziness) at the 4th month of the clinical study. At the follow-up, 5 subjects in the TFC and 7 in the FCT group discontinued the study.

## 4. Discussion

This single-centre, interventional, open-label study revealed that a TFC of antihypertensive drugs was more effective than FCT in LVMI lowering and LVH regression. Observational and prospective studies performed to assess the potential clinical benefits of regression of echocardiographic detectable LVH, found that changes in LVMI during antihypertensive treatment have a strong prognostic impact [7] in terms of cardiovascular and cerebrovascular outcomes. It has also been suggested that a higher cardiovascular risk may persist in patients with LVH regression *vs.* patients with persistently normal LVMI [8]. From a pathophysiological point of view, the better prognosis associated with LVH regression seems to be related to the improvement of systolic and diastolic LV function, with the increase of coronary flow reserve and the decrease of left atrial enlargement and cardiac arrhythmias.

Consequently, the evaluation of changes in LVMI during an antihypertensive treatment adds a prognostic information, particularly in patients with persistence or development of LVH [10]. In this respect, it has been demonstrated that an effective and long-term antihypertensive treatment, ensuring a gradual, constant and homogeneous control of 24 h BP values, may achieve a significant reduction of LVMI, and even a normalization of LVH [12].

The results of the ACCORD (Action to Control Cardiovascular Risk in Diabetes) [13] and SPRINT (Systolic Blood Pressure Intervention Trial) [14] trials have shown that targeting SBP to a more intensive (< 120 mmHg) rather than a less aggressive target (< 140 mmHg) produces a greater reduction in ECG criteria of LVH in hypertensive patients, with and without diabetes mellitus, at high cardiovascular risk. On the other hand, available studies also suggest that for comparable BP reduction, a quicker or a more complete LVH regression may be reached through specific classes of antihypertensive drugs, such as ACEIs, ARBs and CCBs [9,10].

Furthermore, the full normalization of left ventricular geometry is usually observed [21], and echo-reflectivity studies suggest that tissue composition of the left ventricle may vary, and that drugs aiding LVH regression may differently affect myocardial fibrosis [11]. The time course of LVH regression has not been clearly defined, as it may occur over months to years. However, it is commonly agreed that a significant LVMI reduction can only be observed after a 6–8 month-long antihypertensive treatment [22]. In our study, the echocardiographic follow-up has been deliberately lengthened beyond 12 months, with the aim of detecting any significant LVMI reduction and LVH regression, as the mean age of our patients (60.8 ± 12.1 years) and a long history of hypertension could have affected the results of our study in the short term.

In this view, since the BP lowering effect obtained in our study with the TFC and the FCT treatment was comparable, we speculate that the greater LVMI reduction and LVH regression obtained with the TFC treatment is probably related to the “intrinsic” properties of the antihypertensive drugs tested in the TFC group. Due to the lack of face-to-face trials between ACEIs, it is common opinion that they result in similar outcomes in the clinical practice [16]. As a consequence, ACEIs are prescribed interchangeably and deemed to provide the same outcomes for all patients when used chronically [23]. The role of perindopril in LVMI reduction is well known both in human subjects and in animal models [24], and it was found to be more effective than β-blockers in reversing LVH [25]. Indeed, differences between perindopril and enalapril/lisinopril exist and translate into a 2–3-fold greater reduction of major cardiovascular outcomes, as observed in ALLHAT, ASCOT, EUROPA and PROGRESS trials [26–29]. Similarly, in the HOPE study, ramipril was found to affect organ damage and cardiovascular outcomes in high risk hypertensive subjects [30].

As a consequence, it seems evident that perindopril, and to a lesser extent ramipril, have good clinical outcomes, warranting their selection over any other ACEI and in part explaining the positive effects on LVMI reduction and LVH regression observed in our study both in TFC and FCT groups. However, these results should be interpreted with caution. Many of the studies cited above used perindopril in varying dosages and in combination with a variety of other drugs, and thus it was not possible to attribute the beneficial effects achieved in our study solely to ACEI.

Perindopril, alone or in combination with amlodipine and indapamide, has been shown to contribute to the positive effects on cardiovascular outcomes [27,28,31]. Furthermore, the positive effects of indapamide and amlodipine individually may also have contributed to the results on LVMI and LVH observed in this study. In fact, a treatment regimen with amlodipine is associated with a significant reduction in LVMI in hypertensive subjects with mild to moderate essential hypertension [32]. In addition, in the LIVE study [33], indapamide was shown to influence LVMI and even to be significantly more effective than enalapril in reducing LVMI in hypertensive patients with LVH. Furthermore, indapamide reduced LVMI by 17% (P < 0.001), whereas hydrochlothiazide had no significant effect on cardiac organ damage [34].

On the other hand, as observed in the PICXEL study, a combined single-pill antihypertensive regimen based on perindopril and indapamide led to a significant LVMI reduction, as the increased activity of plasma renin induced by the diuretic enhanced the efficacy of the ACEI [35].

The greater LVH regression observed in the FCT group is only in part intuitive as the better BP control does not depend only on the absolute BP reduction, that at follow- up resulted to be similar in the two treatment groups. The better BP control reached in the FCT group particularly depends on the higher modulation of 24 h-BPV, that has been shown to be related to drug-induced regression of target-organ damage at cardiac level. Furthermore, several studies have suggested that BPV is independently associated with the occurrence of LVH and of cardiovascular and renal events [36]. Finally, BPV calculated by 24 h-ABPM has been shown to be associated to LVH independently of mean BP values [37].

In another study, after a follow-up period of 7 years, baseline BPV was found to contribute to the development of cardiovascular complications, particularly LVH [38]. Daytime systolic BPV estimated by SD obtained from 24 h-ABPM has been found to be associated with increased vascular damage and LVH in over 700 subjects with normotension or hypertension of different degrees of severity [39]. Therefore, BPV reduction has been suggested as an additional target in the treatment of cardiovascular diseases [40].

Evidence obtained from meta-analyses and controlled clinical trials has shown that various classes of antihypertensive drugs differ in their ability to control excessive BP fluctuations, with a heterogeneous impact in the prevention of cardiovascular events [40]. CCBs seem to be more effective than other drugs in BP lowering [41], and amlodipine in particular seems to be even more effective than other CCBs in reduction of short-term and long-term BPV [42]. More specifically, in the ASCOTBPLA trial [27], the SBP SD was lower in the amlodipine group than in the atenolol group at all the follow-up visits, due to lower intra visit-to-visit variability. In addition, short-term BPV, intra-visit and 24 h-ABPM variability in SBP was also lower in the amlodipine group than in the atenolol group. When compared with baseline values, while BPV was reduced in the amlodipine group, atenolol treatment was associated with opposite effects. Interestingly, the amlodipine group showed a lower risk of stroke and coronary events with respect to subjects assigned to atenolol treatment, but this beneficial effect was abolished after adjustment for intra-individual BPV [43].

Webb et al. also reviewed the effect of different classes of antihypertensive drugs on BPV in clinical trials [44]. BPV was effectively reduced by CCBs, while drugs acting on the RAAS, thiazide-type diuretics (*e.g.* indapamide), and β-blockers were the least effective and showed neutral effects in comparison with placebo. We speculate that the significant reduction in BPV observed with TFC compared with FTC therapy is probably due to the higher prevalence of patients taking amlodipine in the TFC group (100% *vs*. 70%).

## 5. Study Limitations

Our study was monocentric and limited by its modest cohort size and open-label administration of medications. In addition, the FCT combination was different from the single-pill tested in the TFC group. A large-scale study will be necessary to confirm the effect on LVMI and LVH of the TFC therapy we tested *vs.* a free-combination therapy.

## 6. Conclusion

Patients with LVH at baseline and in whom LVMI reduction has not been reached during the antihypertensive treatment should be considered at high risk for cardiovascular events. Therefore, they should undergo frequent and accurate clinical controls for BP. LVH regression represents the most clinically useful intermediate end-point for the evaluation of the efficacy and of the protective effect of antihypertensive treatment on the cardiovascular system. Our study showed that a TFC of perindopril/indapamide/amlodipine was effective in reducing LVMI and in regressing LVH in patients with moderate hypertension, uncontrolled by a dual fixed-combination therapy. The effectiveness of TFC treatment was probably related to the intrinsic properties of the drugs and to a better control of BP stability. No major concern with TFC and FCT tolerability emerged in this study.

Finally, as recommended in the last ESC/ESH Hypertension guidelines [45], our findings support the choice by clinicians of a fixed-dose antihypertensive drug combination in a single-pill over a free-combination therapy to improve the cardiac organ damage.

## Figures and Tables

**Table 1:** Baseline characteristics.

	All patients (N = 184)	FCT (N = 92)	TFC (N = 92)	p-value
Age (years)	60.8 ± 12.1	61.4 ± 11.8	60.1 ± 12.5	NS
Men (%)	58.0	56.8	65.0	NS
Body mass index (kg/m^2^)	28.5 ± 4.9	28.1 ± 4.7	28.9 ± 5.1	NS
Obesity (%)	26.1	29.3	22.6	NS
	*Clinic BP values*			
SBP (mmHg)	153.7 ± 12.6	153.2 ± 11.8	154.6 ± 13.0	NS
DBP (mmHg)	94.5 ± 6.0	95.1 ± 5.3	94.0 ± 6.7	NS
PP (mmHg)	59.2 ± 11.2	58.1 ± 10.3	60.6 ± 12.1	NS
	*24 h-ABPM values*			
24 h-SBP (mmHg)	144.2 ± 9.7	144.8 ± 8.9	143.6 ± 10.8	NS
24 h-DBP (mmHg)	86.0 ± 6.9	85.3 ± 7.2	86.8 ± 6.5	NS
24 h-PP (mmHg)	58.1 ± 7.6	59.5 ± 7.2	56.8 ± 8.1	NS
Daytime SBP (mmHg)	147.5 ± 9.8	147.2 ± 9.1	148.0 ± 10.9	NS
Daytime DBP (mmHg)	91.7 ± 6.3	92.4 ± 5.5	90.1 ± 7.1	NS
Daytime PP (mmHg)	55.8 ± 7.4	54.8 ± 7.9	57.8 ± 8.3	NS
Night-time SBP (mmHg)	129.6 ± 10.0	128.9 ± 9.3	130.5 ± 10.5	NS
Night-time DBP (mmHg)	77.0 ± 6.8	77.6 ± 7.3	76.4 ± 6.1	NS
Night-time PP (mmHg)	562.6 ± 6.3	51.3 ± 5.9	54.1 ± 7.2	NS
LVH (%)	70.1	68.5	71.7	NS
LVEF (%)	62.5	64.3	58.1	NS
TC (mg/dl)	198.1 ± 23.6	200.2 ± 18.3	196.8 ± 29.3	NS
LDL-C (mg/dl)	123.5 ± 23.6	124.3 ± 21.2	122.8 ± 26.9	NS
HDL-C (mg/dl)	52.9 ± 13.0	51.4 ± 12.7	53.3 ± 13.8	NS
Triglycerides (mg/dl)	101.4 ± 40.2	99.7 ± 33.5	103.4 ± 46.8	NS
Glucose (mg/dl)	99.7 ± 11.3	98.4 ± 8.3	100.8 ± 14.4	NS
Creatinine (mg/dl)	1.0 ± 0.2	1.1 ± 0.1	1.0 ± 0.3	NS
eGFR (ml/min/1,73 m^2^)	90.9 ± 32.8	91.4 ± 33.3	90.5 ± 33.6	NS
Potassium (mg/dl)	4.28 ± 0.27	4.17 ± 0.31	4.36 ± 0.22	NS
Diabetes (%)	11.9	10.2	13.6	NS
Smokers (%)	19.8	17.0	20.1	NS

24 h-ABPM: 24 h ambulatory blood pressure monitoring; DBP: diastolic blood pressure; FCT: free combination therapy; HDL-C high-density-lipoprotein; LDL-C low-density-lipoprotein cholesterol, LVH: left ventricular hypertrophy; LVEF: left ventricular ejection fraction; PP: pulse pressure, SBP systolic blood pressure, TFC: tripled fixed combination; TC total cholesterol. Heart rate was not different between treatment groups.

**Table 2:** Office blood pressure (BP) and 24-h ambulatory blood pressure monitoring values from baseline to follow-up, in the group treated with tripled fixed-combination of anti-hypertensive drugs.

	Baseline (n = 92)	1st month (n = 92)	4th month (n = 89)	14th month (n = 84)	p-value for trend
	*Office BP values*				
SBP (mmHg)	154.6 ± 13.0	139.3 ± 8.1	132.2 ± 5.1	130.8 ± 4.6	0.0001
DBP (mmHg)	94.0 ± 7.0	84.5 ± 6.7	82.9 ± 4.8	81.2 ± 3.3	0.0001
PP (mmHg)	60.6 ± 12.1	54.8 ± 7.3	50.3 ± 5.6	49.6 ± 4.4	0.0001
HR (bpm)	74.3 ± 6.9	73.3 ± 5.9	74.2 ± 4.5	74.4 ± 4.1	0.246
	*24 h-ABPM values*				
24 h-SBP (mmHg)	143.6 ± 10.8	132.0 ± 8.1	125.5 ± 5.6	125.1 ± 5.1	0.001
24 h-DBP (mmHg)	86.8 ± 6.5	79.8 ± 4.6	77.9 ± 3.9	78.2 ± 4.0	0.0001
24 h-PP (mmHg)	56.8 ± 8.1	52.2 ± 6.7	50.6 ± 3.4	46.9 ± 4.6	0.001
24 h-HR (bpm)	72.7 ± 6.6	72.9 ± 6.3	73.2 ± 4.5	72.7 ± 4.7	0.528
Daytime SBP (mmHg)	148.0 ± 10.9	132.0 ± 8.1	128.5 ± 3.6	128.2 ± 3.9	0.0001
Daytime DBP (mmHg)	90.1 ± 7.1	83.1 ± 4.9	78.0 ± 3.9	78.3 ± 4.3	0.0001
Daytime PP (mmHg)	57.8 ± 8.3	52.2 ± 6.7	50.6 ± 3.4	49.9 ± 3.7	0.0001
Daytime HR (bpm)	75.1 ± 6.7	72.9 ± 6.3	73.2 ± 4.6	74.5 ± 5.3	0.410
Night-time SBP (mmHg)	130.5 ± 10.5	119.1 ± 7.7	116.1 ± 4.0	117.4 ± 4.2	0.0001
Night-time DBP (mmHg)	76.4 ± 6.1	69.3 ± 4.9	66.1 ± 3.8	66.3 ± 3.2	0.0001
Night-time PP (mmHg)	54.1 ± 7.2	50.0 ± 5.9	50.3 ± 3.6	51.1 ± 4.0	0.0001
Night-time HR (bpm)	67.0 ± 6.6	66.5 ± 5.4	68 ± 3.9	67.6 ± 4.3	0.394

24 *h-ABPM:* 24 h ambulatory blood pressure monitoring, *DBP* diastolic blood pressure, *PP* pulse pressure, *SBP* systolic blood pressure; *HR*: heart rate.

**Table 3:** Office blood pressure (BP) and 24-h ambulatory blood pressure monitoring values from baseline to follow-up, in the group treated with the free combination of three antihypertensive drugs.

	Baseline (n = 92)	1st month (n = 92)	4th month (n = 90)	14th month (n = 83)	p-value by trend
	*Office BP values*				
SBP (mmHg)	153.2 ± 11.8	140.1 ± 7.3	134.3 ± 4.1	131.2 ± 2.5	0.0001
DBP (mmHg)	95.1 ± 5.3	85.4 ± 8.3	83.4 ± 5.6	82.4 ± 3.4	0.0001
PP (mmHg)	58.1 ± 10.3	56.7 ± 6.1	50.9 ± 4.8	48.8 ± 3.8	0.0001
HR (bpm)	73.4 ± 8.1	74.8 ± 7.3	75.2 ± 5.3	75.7 ± 4.4	0.582
	*24 h-ABPM values*				
24 h-SBP (mmHg)	144.8 ± 8.9	137.9 ± 7.5	127.3 ± 6.4	126.5 ± 5.2	0.001
24 h-DBP (mmHg)	85.3 ± 7.2	81.8 ± 6.3	78.6 ± 4.8	78.0 ± 4.1	0.0001
24 h-PP (mmHg)	59.5 ± 7.2	56.1 ± 6.7	48.7 ± 5.1	48.5 ± 4.8	0.001
24 h-HR (bpm)	78.9 ± 8.2	80.1 ± 6.9	79.6 ± 5.7	78.2 ± 5.3	0.324
Daytime SBP (mmHg)	147.2 ± 9.1	141.7 ± 5.9	130.0 ± 4.8	129.5 ± 4.0	0.0001
Daytime DBP (mmHg)	92.4 ± 5.5	85.8 ± 5.7	81.2 ± 5.7	80.4 ± 5.0	0.0001
Daytime PP (mmHg)	54.8 ± 7.9	55.9 ± 7.4	48.8 ± 4.1	49.1 ± 3.9	0.0001
Daytime HR (bpm)	82.3 ± 6.8	79.4 ± 5.6	80.6 ± 6.2	79.2 ± 5.7	0.156
Night-time SBP (mmHg)	128.9 ± 9.3	124.1 ± 8.2	118.2 ± 7.4	119.6 ± 6.8	0.0001
Night-time DBP (mmHg)	77.6 ± 7.3	70.4 ± 6.1	69.1 ± 8.2	68.9 ± 7.6	0.0001
Night-time PP (mmHg)	51.3 ± 5.9	53.7 ± 3.6	49.1 ± 4.3	50.7 ± 3.9	0.0001
Night-time HR (bpm)	70.2 ± 7.8	69.7 ± 6.2	71.6 ± 5.7	70.3 ± 5.3	0.418

24 *h-ABPM:* 24 h ambulatory blood pressure monitoring, *DBP* diastolic blood pressure, *PP* pulse pressure, *SBP* systolic blood pressure; *HR*: heart rate.

## References

[1] Cuspidi C, Rescaldani M, Sala C, Negri F, Grassi G, Mancia G, Prevalence of electrocardiographic left ventricular hypertrophy in human hypertension: an updated review, J. Hypertens 30 (11 11) (2012) 2066–2073. 22914541 10.1097/HJH.0b013e32835726a3

[2] Regitz-Zagrosek V, Oertelt-Prigione S, Prescott E, Franconi F, Gerdts E, Foryst-Ludwig A, , Gender in cardiovascular diseases: impact on clinical manifestations, management, and outcomes, Eur. Heart J 37 (1 1) (2016) 24–34. 26530104 10.1093/eurheartj/ehv598

[3] Lang RM, Badano LP, Mor-Avi V, Afilalo J, Armstrong A, Ernande L, , Recommendations for cardiac chamber quantification by echocardiography in adults: an update from the American Society of Echocardiography and the European Association of Cardiovascular Imaging, Eur. Heart J. Cardiovasc. Imaging 16 (3 3) (2015) 233–270. 25712077 10.1093/ehjci/jev014

[4] Muiesan ML, Salvetti M, Di Castelnuovo A, Paini A, Assanelli D, Costanzo S, , Obesity and ECG left ventricular hypertrophy, J. Hypertens 35 (1 1) (2017) 162–169. 27662187 10.1097/HJH.0000000000001121

[5] Bang CN, Devereux RB, Okin PM, Regression of electrocardiographic left ventricular hypertrophy or strain is associated with lower incidence of cardiovascular morbidity and mortality in hypertensive patients independent of blood pressure reduction. A LIFE review, J. Electrocardiol 47 (Sep-Oct 5) (2014) 630–635. 25052475 10.1016/j.jelectrocard.2014.07.003

[6] Angeli F, Reboldi G, Poltronieri C, Stefanetti E, Bartolini C, Verdecchia P, , The prognostic legacy of left ventricular hypertrophy: cumulative evidence after the MAVI study, J. Hypertens 33 (11 11) (2015) 2322–2330. 26335428 10.1097/HJH.0000000000000692

[7] Devereux RB, Wachtell K, Gerdts E, Boman K, Nieminen MS, Papademetriou V, , Prognostic significance of left ventricular mass change during treatment of hypertension, JAMA 292 (11 19) (2004) 2350–2356. 15547162 10.1001/jama.292.19.2350

[8] Muiesan ML, Salvetti M, Paini A, Monteduro C, Galbassini G, Bonzi B, , Inappropriate left ventricular mass changes during treatment adversely affects cardiovascular prognosis in hypertensive patients, Hypertension. 49 (5 5) (2007) 1077–1083. 17372030 10.1161/HYPERTENSIONAHA.107.087320

[9] Devereux RB, Dahlöf B, Gerdts E, Boman K, Nieminen M, Papademetriou V, , Regression of hypertensive left ventricular hypertrophy by losartan compared with atenolol: the Losartan Intervention for Endpoint Reduction in Hypertension (LIFE) trial, Circulation 110 (9 11) (2004) 1456–1462. 15326072 10.1161/01.CIR.0000141573.44737.5A

[10] Fagard RH, Celis H, Thijs L, Wouters S, Regression of left ventricular mass by antihypertensive treatment: a meta-analysis of randomized comparative studies, Hypertension 54 (11 5) (2009) 1084–1091. 19770405 10.1161/HYPERTENSIONAHA.109.136655

[11] Ciulla MM, Paliotti R, Esposito A, Dìez J, López B, Dahlöf B, , Different effects of antihypertensive therapies based on losartan or atenolol on ultrasound and biochemical markers of myocardial fibrosis: results of a randomized trial, Circulation 110 (8 5) (2004) 552–557. 15277331 10.1161/01.CIR.0000137118.47943.5C

[12] Gerc V, Begović B, Vehabović M, Georgievich Voronkov L, Vataman E, Musić L, Buksa M, , Effects of fixed combination of lisinopril plus hydrochlorothiazide on regression of left ventricular hypertrophy in patients with essential hypertension: an opened, multi-centre, prospective clinical trial, Bosn. J. Basic Med. Sci 8 (8 3) (2008) 214–219. 18816251 10.17305/bjbms.2008.2920 PMC5694670

[13] Soliman EZ, Byington RP, Bigger JT, Evans G, Okin PM, Goff Jret al DC., Effect of intensive blood pressure lowering on left ventricular hypertrophy in patients with diabetes mellitus: action to control cardiovascular risk in diabetes blood pressure trial, Hypertension 66 (12 6) (2015) 1123–1129. 26459421 10.1161/HYPERTENSIONAHA.115.06236 PMC4644090

[14] Soliman EZ, Ambrosius WT, Cushman WC, Zhang ZM, Bates JT, Neyra JA, , Effect of intensive blood pressure lowering on left ventricular hypertrophy in patients with hypertension: the systolic blood pressure intervention (SPRINT) trial, Circulation 136 (8 5) (2017) 440–450. 28512184 10.1161/CIRCULATIONAHA.117.028441 PMC5538944

[15] Mazza A, Lenti S, Schiavon L, Sacco AP, Dell’Avvocata F, Rigatelli G, , Fixed-dose triple combination of antihypertensive drugs improves blood pressure control: from clinical trials to clinical practice, Adv. Ther 34 (4 4) (2017) 975–985. 28299716 10.1007/s12325-017-0511-1

[16] Mancia G, Fagard R, Narkiewicz K, Redón J, Zanchetti A, Böhm M, , ESH/ESC Guidelines for the management of arterial hypertension: the task force for the management of arterial hypertension of the European Society of Hypertension (ESH) and of the European Society of Cardiology (ESC), J. Hypertens 31 (7 7) (2013) 1281–1357 2013. 23817082 10.1097/01.hjh.0000431740.32696.cc

[17] Palatini P, Frigo G, Bertolo O, Roman E, Da Corta R`, Winnicki M, Validation of the A&D TM-2430 device for ambulatory blood pressure monitoring and evaluation of performance according to subject’s characteristics, Blood Press. Monit 3 (8 4) (1998) 255–260. 10212363

[18] Cockcroft DW, Gault MH, Prediction of creatinine clearance from serum creatinine, Nephron 16 (1976) 31–41. 1244564 10.1159/000180580

[19] Sahn DJ, De Maria A, Kissio J, Weyman A, The committee on m-mode standardization of the American society of echocardiography. Recommendations regarding quantitation in M-mode echocardiography: results of a survey of echocardiographic measurements, Circulation 58 (12 6) (1978) 1072–1083. 709763 10.1161/01.cir.58.6.1072

[20] Devereux RB, Reichek N, Echocardiographic determination of mass in man: anatomic validation of the method, Circulation 55 (1977) 613–618. 138494 10.1161/01.cir.55.4.613

[21] Wachtell K, Palmieri V, Olsen MH, Gerdts E, Papademetriou V, Nieminen MS, , Change in systolic left ventricular performance after 3 years of antihypertensive treatment: the Losartan Intervention for Endpoint (LIFE) Study, Circulation 106 (7 2) (2002) 227–232. 12105163 10.1161/01.cir.0000021601.49664.2a

[22] Cuspidi C, Tadic M, Grassi G, Mancia G, Treatment of hypertension: the ESH/ESC guidelines recommendations, Pharmacol. Res 128 (Febuary) (2018) 315–321. 29080798 10.1016/j.phrs.2017.10.003

[23] Furberg CD, Pitt B, Are all angiotensin-converting enzyme inhibitors interchangeable? J. Am. Coll. Cardiol 37 (4 5) (2001) 1456–1460. 11300461 10.1016/s0735-1097(01)01161-5

[24] Gosse P, Grellet J, Bonoron S, Tariosse L, Besse P, Dallocchio M, Effects of perindopril on left ventricular hypertrophy, coronary blood flow, and mechanical properties of cardiac muscle in renovascular hypertensive rats, Am. J. Hypertens 4 (3 3 Pt 2) (1991) 235S–239S. 1828352 10.1093/ajh/4.3.235s

[25] Hui Y, Dai Z, Chen X, Wang W, Effect of perindopril and metoprolol on left ventricular hypertrophy and performance in essential hypertension, Chin. Med. J 108 (9 9) (1995) 678–681. 8575234

[26] Wright JT, Davis BR, Major outcomes in high-risk hypertensive patients randomised to angiotensin-converting enzyme inhibitor or calcium channel blocker vs. diuretic. The Antihypertensive and Lipid-Lowering Treatment to prevent Heart Attack Trial (ALLHAT), JAMA 288 (12 23) (2002) 2981–2997. 12479763 10.1001/jama.288.23.2981

[27] Dahlöf B, , Prevention of cardiovascular events with an antihypertensive regimen adding perindopril as required versus atenolol adding bendroflumenthiazide as required, in the Anglo- Scandinavian Cardiac Outcomes Trials – blood Pressure lowering Arm (ASCOT-BPLA): a multicentre randomised controlled trial, Lancet 366 (9 9489) (2005) 895–906. 16154016 10.1016/S0140-6736(05)67185-1

[28] Fox KM, , Efficacy of perindopril in reduction of cardiovascular events among patients with stable coronary artery disease: randomised, double-blind, placebo-controlled, multicentre trial (The EUROPA study), Lancet 362 (9 9386) (2003) 782–788. 13678872 10.1016/s0140-6736(03)14286-9

[29] PROGRESS collaborative group, Randomised trial of a perindopril-based blood-pressure-lowering regimen among 6105 individuals with previous stroke or transient ischaemic attack, Lancet 358 (9 9287) (2001) 1033–1041. 11589932 10.1016/S0140-6736(01)06178-5

[30] Yusuf S, , Effects of an angiotensin-converting-enzyme inhibitor, ramipril, on cardiovascular events in high-risk patients (HOPE study), N. Engl. J. Med 342 (1 3) (2000) 145–153. 10639539 10.1056/NEJM200001203420301

[31] ADVANCE collaborative group, Effects of a fixed combination of perindopril and indapamide on macrovascular and microvascular outcomes in patients with type 2 diabetes mellitus (the ADVANCE trial): a randomised controlled trial, Lancet 370 (9 9590) (2007) 829–840. 17765963 10.1016/S0140-6736(07)61303-8

[32] Islim IF, Watson RD, Ihenacho HN, Ebanks M, Singh SP, Amlodipine: effective for treatment of mild to moderate essential hypertension and left ventricular hypertrophy, Cardiology 96 (Suppl. 1) (2001) 10–18. 11574741 10.1159/000049096

[33] Gosse P, Sheridan DJ, Zannad F, Dubourg O, Guéret P, Karpov Y, , Regression of left ventricular hypertrophy in hypertensive patients treated with indapamide SR 1.5 mg versus enalapril 20 mg: the LIVE study, J. Hypertens 18 (10 10) (2000) 1465–1475. 11057435 10.1097/00004872-200018100-00015

[34] Senior R, Imbs JL, Bory M, Amabile G, Denis B, Zannad F, , Indapamide reduces hypertensive left ventricular hypertrophy: an international multicenter study, J. Cardiovasc. Pharmacol 22 (Suppl. 6) (1993) S106–10. 7508055

[35] Dahlöf B, Gosse P, Guéret P, Dubourg O, de Simone G, Schmieder R, , Perindopril/indapamide combination more effective than enalapril in reducing blood pressure and left ventricular mass: the PICXEL study, J. Hypertens 23 (11 11) (2005) 2063–2070. 16208150 10.1097/01.hjh.0000187253.35245.dc

[36] Narkiewicz K, Winnicki M, Schroeder K, Phillips BG, Kato M, Cwalina E, , Relationship between muscle sympathetic nerve activity and diurnal blood pressure profile, Hypertension 39 (1 1) (2002) 168–172. 11799097 10.1161/hy1201.097302

[37] Tatasciore A, Zimarino M, Tommasi R, Renda G, Schillaci G, Parati G, , Increased short-term blood pressure variability is associated with early left ventricular systolic dysfunction in newly diagnosed untreated hypertensive patients, J. Hypertens 31 (8 8) (2013) 1653–1661. 23811997 10.1097/HJH.0b013e328361e4a6

[38] Frattola A, Parati G, Cuspidi C, Albini F, Mancia G, Prognostic value of 24-hour blood pressure variability, J. Hypertens 11 (10 10) (1993) 1133–1137. 8258679 10.1097/00004872-199310000-00019

[39] Palatini P, Penzo M, Racioppa A, Zugno E, Guzzardi G, Anaclerio M, , Clinical relevance of nighttime blood pressure and of daytime blood pressure variability, Archives Inter Med. 152 (9 9) (1992) 1855–1860. 1387782

[40] Olesen TB, Stidsen JV, Blicher MK, Pareek M, Rasmussen S, Vishram-Nielsen JKK, , Impact of age and target-organ damage on prognostic value of 24-Hour ambulatory blood pressure, Hypertension 70 (11 5) (2017) 1034–1041. 28893899 10.1161/HYPERTENSIONAHA.117.09173

[41] Nozato S, Yamamoto K, Nozato Y, Takeda M, Hongyo K, Takeya M, , Comparison between L-type and N/L-type calcium channel blockers in the regulation of home blood-pressure variability in elderly hypertensive patients, Hypertens. Res 41 (4 4) (2018) 290–298. 29449705 10.1038/s41440-018-0018-4

[42] Zhang L, Yang J, Li L, Liu D, Xie X, Dong P, , Comparison of amlodipine versus other calcium channel blockers on blood pressure variability in hypertensive patients in China: a retrospective propensity score-matched analysis, J. Comp. Eff. Res 7 (7 7) (2018) 651–660. 29888950 10.2217/cer-2017-0063

[43] Hocht C, Bertera FM, Taira CA, Importance of blood pressure variability in the assessment of cardiovascular risk and benefits of antihypertensive therapy, Expert Rev. Clin. Pharmacol 3 (9 5) (2010) 617–621. 22111743 10.1586/ecp.10.44

[44] Webb AJ, Fischer U, Mehta Z, Rothwell PM, Effects of antihypertensive-drug class on interindividual variation in blood pressure and risk of stroke: a systematic review and metaanalysis, Lancet 375 (3 9718) (2010) 906–915. 20226989 10.1016/S0140-6736(10)60235-8

[45] Williams B, Mancia G, Spiering W, Agabiti Rosei E, Azizi M, Burnier M, , Practice guidelines for the management of arterial hypertension of the european society of hypertension and the european society of cardiology: ESH/ESC task force for the management of arterial hypertension, J. Hypertens 36 (12 12) (2018) 2284–2309 2018. 30379783 10.1097/HJH.0000000000001961

